# Small extracellular vesicle-mediated metabolic reprogramming: from tumors to pre-metastatic niche formation

**DOI:** 10.1186/s12964-023-01136-x

**Published:** 2023-05-19

**Authors:** Chuwen Jiang, Zhengting Jiang, Gengyu Sha, Daorong Wang, Dong Tang

**Affiliations:** 1 Clinical Medical College, Yangzhou University, Yangzhou, 225001 Jiangsu Province China; 2 Department of General Surgery, Institute of General Surgery, Clinical Medical College, Yangzhou University, Northern Jiangsu People’s Hospital, Yangzhou, 225001 China

**Keywords:** sEVs, Metabolic reprogramming, Tumor microenvironment, PMN, Metastasis

## Abstract

Metastasis, the spread of a tumor or cancer from the primary site of the body to a secondary site, is a multi-step process in cancer progression, accounting for various obstacles in cancer treatment and most cancer-related deaths. Metabolic reprogramming refers to adaptive metabolic changes that occur in cancer cells in the tumor microenvironment (TME) to enhance their survival ability and metastatic potential. Stromal cell metabolism also changes to stimulate tumor proliferation and metastasis. Metabolic adaptations of tumor and non-tumor cells exist not only in the TME but also in the pre-metastatic niche (PMN), a remote TME conducive for tumor metastasis. As a novel mediator in cell-to-cell communication, small extracellular vesicles (sEVs), which have a diameter of 30–150 nm, reprogram metabolism in stromal and cancer cells within the TME by transferring bioactive substances including proteins, mRNAs and miRNAs (microRNAs). sEVs can be delivered from the primary TME to PMN, affecting PMN formation in stroma rewriting, angiogenesis, immunological suppression and matrix cell metabolism by mediating metabolic reprogramming. Herein, we review the functions of sEVs in cancer cells and the TME, how sEVs facilitate PMN establishment to trigger metastasis via metabolic reprogramming, and the prospective applications of sEVs in tumor diagnosis and treatment.

Video Abstract

**Supplementary Information:**

The online version contains supplementary material available at 10.1186/s12964-023-01136-x.

## Background

Tumor metastasis is a process whereby tumor cells migrate from a primary site to distant organs and tissues progressively, resulting in the majority deaths among tumor patients [1]. The establishment of the pre-metastasis niche (PMN), a favorable tumor microenvironment (TME) in a distant metastatic organ, is a prerequisite to sustain the remote settlement of tumor cells and accelerate metastasis [2]. TME is a highly complicated and heterogeneous ecosystem that is composed of tumor cells, fibroblasts, adipocyte cells, endothelial cells (ECs), mesenchymal stem cells (MSCs), and extracellular matrix [3]. Extensive communication between cancer cells and other stromal cells including autocrine and paracrine signal transduction in TME could regulate apoptosis inhibition, immunosuppression, angiogenesis, and metabolic reprogramming, thereby affecting tumor malignant progression like metastasis [3, 4].

Metabolic reprogramming refers to the process wherein the glucose, lipid and amino acid metabolism of cancer cells change adaptively to help them quickly proliferate and survive under the high pressure of environment whether in the primary TME or PMN [5]. As tumor cells reprogram metabolism in every step of tumor progression, metabolic reprogramming can be regarded as a core hallmark of cancer [6]. In a TME, the metabolism of non-cancer cells is also impacted by cancer cells and facilitates cancer cell growth and metastasis in return [3]. Small extracellular vesicles (sEVs), which have a diameter of 30–150 nm, as one of three subtypes of extracellular vesicles, mediate intracellular communication in TME [7]. As influential constitutes in TME, sEVs are released by various cells (containing tumor and stromal cells), via multivesicular bodies to enhance the crosstalk between these cells and induce metabolic changes in them by transferring biologically active constituents including protein, mRNA, and miRNA to recipient cells [3, 8]. Except for local signaling within the primary TME, sEVs may circulate to spread the “seeds” that qualify the “soil” at distant niches to facilitate metastasis [9]. Owing to their abundant cargoes, sEVs from animal cells, body fluids and even plant cells are used for ideal carriers of substances or drugs to target tumor metabolism, inhibiting tumor progression [10].

Herein, we review how sEVs mediate the metabolism of tumor and stromal cells in TME and promote the PMN formation via metabolic reshaping as well as their potential applications in tumor diagnosis and anti-tumor therapy.

## sEVs are involved in the metabolic reprogramming of cancer cells

The metabolic process of any cell, including cancer cells, is inseparable from the function of crucial enzymes. Many key oncogenic signaling pathways and factors such as KRAS, inositol phospho3-kinase/AKT (PI3K/Akt), c-Myc, p53, and hypoxia-inducible factor-1 (HIF-1) influence the regulation of cancer cell metabolism [11]. sEVs as the significant medium of intercellular communication, secrete bioactive substances to interact with key metabolic enzymes as well as carcinogenic signaling pathways and factors, thus providing a potential mechanism for metabolic reprogramming of tumor cells. Furthermore, metabolic changes in cancer cells were reported to influence sEV secretion to promote lung cancer metastasis [12].

### sEVs regulate glucose metabolic reprogramming in cancer cells

**Fig. 1:**
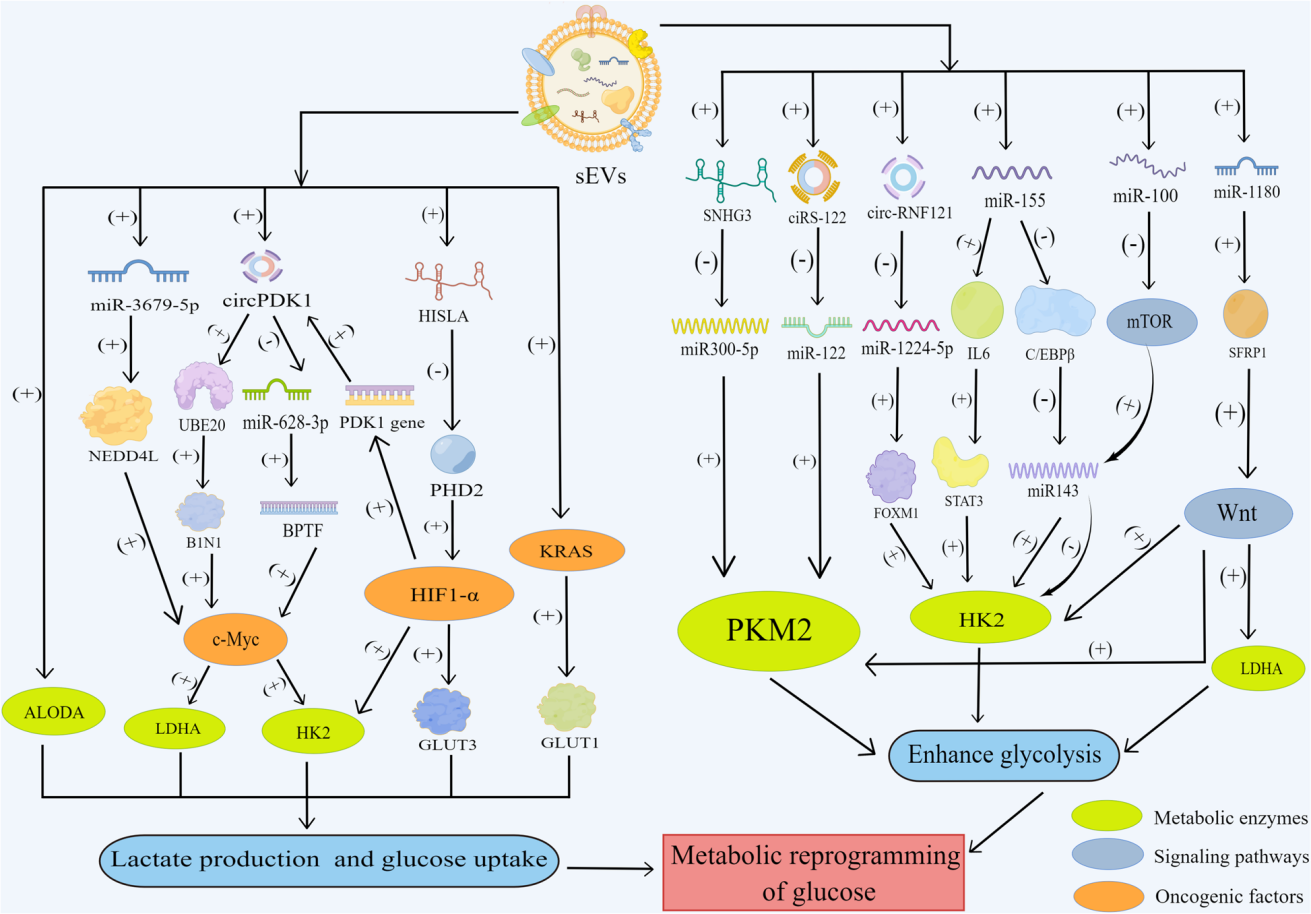
sEV-mediated metabolic reprogramming of glucose. The figure was created using Figdraw (www.figdraw.com). ALODA: Aldolase A; LDHA: Lactate dehydrogenase A; HK2: Hexokinase 2; GLUT: Glucose transporter; HIF-1α: Hypoxia-inducible factor-1α; PKM2: Pyruvate kinase M2; mTOR: Mammalian target of rapamycin; HISLA: HIF-1α-stabilizing lncRNA; SNHG3: Small nucleolar RNA host gene 3; STAT3: Signal transducer and activator of transcription 3

Glycolysis or aerobic glycolysis is increased in tumors to facilitate quick proliferation, and helps cancer cells subsist under the high pressures, which is called the “Warburg effect” [5]. sEVs act on glycolytic-related enzymes, oncogenic factors and signaling pathways, enhancing the Warburg effect of tumors (Fig. 1).

#### sEVs interact with oncogenic factors to reshape cancer cell metabolism

The oncogenic transcription factor c-Myc and HIF-1 are primary metabolic reprogramming proteins [5]. c-Myc is a crucial controller of several glycolytic genes by directly increasing the expressions of hexokinase 2 (HK2), lactate dehydrogenase A (LDHA) and others to enhance aerobic glycolysis [13]. HIF-1 serves as another critical factor modulating glucose metabolism, contributing to the genetic transcription associated with glucose transporter (GLUT) and enzymes associated with glycolysis including HK and pyruvate kinase M (PKM) [14]. sEVs enhance the stability of c-Myc and HIF-1 through their secretomes including miRNA and long non-coding RNA (lncRNA), thus reshaping cancer cell metabolism.

NEDD4L, a well-known regulator dominating the stabilization of c-Myc and M2 macrophage-derived sEVs, delivers overexpressed miR-3679-5p to down-regulate the levels of NEDD4L in lung tumor cells, hence enabling the stabilization of c-Myc, which can enhance the levels of HK2 and LDHA and promote glycolysis. M2 macrophage-derived sEVs also mediate this signaling axis to induce chemoresistance in lung cancer via metabolism controlling [15]. Tumor-associated macrophages (TAMs) increase glycolysis and apoptotic resistance in tumor cells through sEVs. Mechanistically, sEV-packaged HIF-1α-stabilizing lncRNA (HISLA) suppresses the function of PHD2 to block the degradation of HIF-1α, whose expression is positively related with the expression levels of GLUT1, GLUT3 and HK2, thus allowing the ability of cancer cells to metabolize glucose in a condition without oxygen to survive for an extended period of time [16]. Interestingly, lactic acid produced by tumor aerobic glycolysis was found to increase the expression of HISLA in TAMs, which further improved glucose metabolism ability of cancer cells, thereby producing the integrated feedback loop between TAMs and tumor cells [16]. Indeed, HIF-1α could influence c-Myc by inducing the high expression of exosome cargos. HIF-1α upregulated circPDK1 expression under the hypoxia condition by transcriptionally activating its host gene PDK1 and hypoxia-induced exosome circPDK1 sponged miR-628-3p to release BPTF, which is required for c-Myc transcriptional activity, indicating that circPDK1 activated the BPTF/c-Myc axis to enhance pancreatic cancer (PC) glycolysis by downregulating miR-628-3p expression. Besides, circPDK1 functioned as a frame to promote the binding of BIN1 proteins (a tumor suppressor limiting c-Myc transcriptional activity) and UBE2O (a ubiquitin-conjugating enzyme), enhancing the effects of UBE2O on ubiquitin-dependent degradation of BIN1 and increasing c-Myc transcriptional activity eventually. As the shared goal of two no cross-talk pathways activated by HIF1α-reduced exosome circPDK1, c-Myc enhanced the expression of glycolytic genes LDHA to promote aerobic glycolysis and migration. In all, HIF-1α upregulated circPDK1, which stimulated c-Myc by regulating miR-628-3p/BPTF signaling and lowering BIN1 to enhance glycolysis and tumor progression [17]. These findings suggest that the interaction between sEVs and carcinogenic factors reprograms tumor cell metabolism and further enhances tumor survival ability as well as promotes cancer progression.

#### sEVs act on key glycolytic proteins and enzymes that regulate cancer glucose metabolism

sEVs act on glucose transporters and abundant enzymes to strikingly influence the metabolism of cancer cells. GLUT1 switches on the first step of glycolysis by transporting glucose to the cytoplasm for glucose metabolism. Mutant KRAS sEVs contain overexpressed GLUT-1, contributing to enhanced glucose absorption and the acquisition of the metabolic change [18]. HK is the first rate-limiting enzyme in the glucose metabolism. HK2 has been identified as a key participant in the Warburg effect and put forward as a metabolic target for cancer therapy owing to its solid position as a major isoenzyme that is overexpressed in tumors [19]. IL-6 is a pro-inflammatory cytokine and activator of signal transducer and activator of transcription 3 (STAT3, a transcriptional activator for HK2). Chronic inflammation is a main backer to the occurrence of cancers and pro-inflammatory cytokine IL6 could boost glycolysis in cancer cells via serum sEV-derived miR-155 [20, 21]. Mechanistically, exosome miR-155 turns on the STAT3 switch through the up-regulation of IL-6 to promote HK2 transcription to enhance aerobic glycolysis. Besides, miR-155 was demonstrated to inhibit mir-143, an adverse manager of HK2, via pointing at C/EBPβ (a transcriptional activator for mir-143), thereby leading to the up-regulation of HK2 at the post-transcriptional level to realize metabolic reprogramming [21].

PKM2 is a subtype of PKM, another rate-limiting enzyme in glycolysis. MiR-122 effectively suppresses protein and mRNA levels of PKM2 expression to reduce lactate production significantly [22]. sEVs carry ciRS-122 to sensitive cells to enhance glycolysis and oxaliplatin resistance via miR-122 sponge targeting PKM2. Accordingly, exosome transport of si-ciRS-122 could alter drug resistance by controlling the ciRS-122-miR-122-PKM2 axis in vivo [23]. The sEV-packaged circ-RNF121 was found to enhance the expressions of HK2 and PKM2 via the down-regulation of miR-1224-5p and the up-regulation of FOXM1 in colorectal cancer (CRC) [24]. Thus, circ-RNF121 regulates tumor glucose metabolism and progression via miR-1224-5p/FOXM1 axis in tumor tissues. The sEV-derived lncRNA small nucleolar RNA host gene 3 (SNHG3) could participate in metabolic changes within tumor cells after absorbing sEVs. SNHG3 knockdown suppressed breast tumor growth by the increased expression of miR-330-5p and decreased expression of PKM. SNHG3 is a molecular sponge for miR-330-5p in breast cancer cells to positively control PKM levels, thus suppressing oxidative phosphorylation (OXPHOS) and enhancing glycolysis to promote cancer proliferation and progression [25]. Except for HK2 and PKM2, the major enzyme aldolase A(ALDOA) transferred by sEVs may act as vital signal transduction elements to regulate the motility of recipient cells and promote metastasis by accelerating the glycolysis process [26]. These results showed amply that the complex interplay between sEVs and crucial protein and enzymes modifies the cancer cells glucose metabolism to enhance tumor migration.

#### sEVs activate signaling pathways in cancer cells that regulate cancer glucose metabolism

sEVs reprogram metabolism in cancer cells not only by acting on enzymes associated with glycolysis but also through the activation of different signaling pathways. KRAS mutations occur in various cancer types and mutant KRAS sEVs comprise overexpressed GLUT-1 to facilitate glycolysis [18]. As previously mentioned, the STAT3 pathway can increase the expression of HK2, inducing the inflammatory environment supporting cancerogenesis and exosome miR-155 boosts the expression of STAT3 [21]. The mammalian target of rapamycin (mTOR) pathway is a crucial pivot in cancer promotion and the mTOR-miR-143/HK2 axis could accelerate tumor proliferation and formation by promoting glucose metabolism in lung cancer [27]. sEV-derived miR-100 could directly inhibit mTOR and eliminate its inhibitory effect on miR-143 (a negative regulator of HK2) to increase the level of miR-143. Meanwhile, miR-143 may be increased directly through sEVs transfer. The miR-143 high expression downregulates the downstream key molecules of the mTOR signaling pathway including HK2 and KRAS, thus influencing cancer cells metabolism reprogramming [28]. Besides, Wnt signaling up-regulated LDHA,HK2 and PKM2 to enhance glycolysis in cancer cells and bone marrow mesenchymal stem cells (BM-MSCs) activated Wnt signaling in tumors via targeting SFRP1 due to the delivery of BM-MSC-derived exosome miR-1180 [29]. In conclusion, sEVs can rewrite the glucose metabolism of cancer cells through interactions with glycolytic-related enzymes, oncogenic factors and oncogenic signaling pathways, providing sufficient energy for tumor proliferation and metastasis.

### sEVs modulate the reprogramming of fatty acid metabolism in cancer cells

**Fig. 2:**
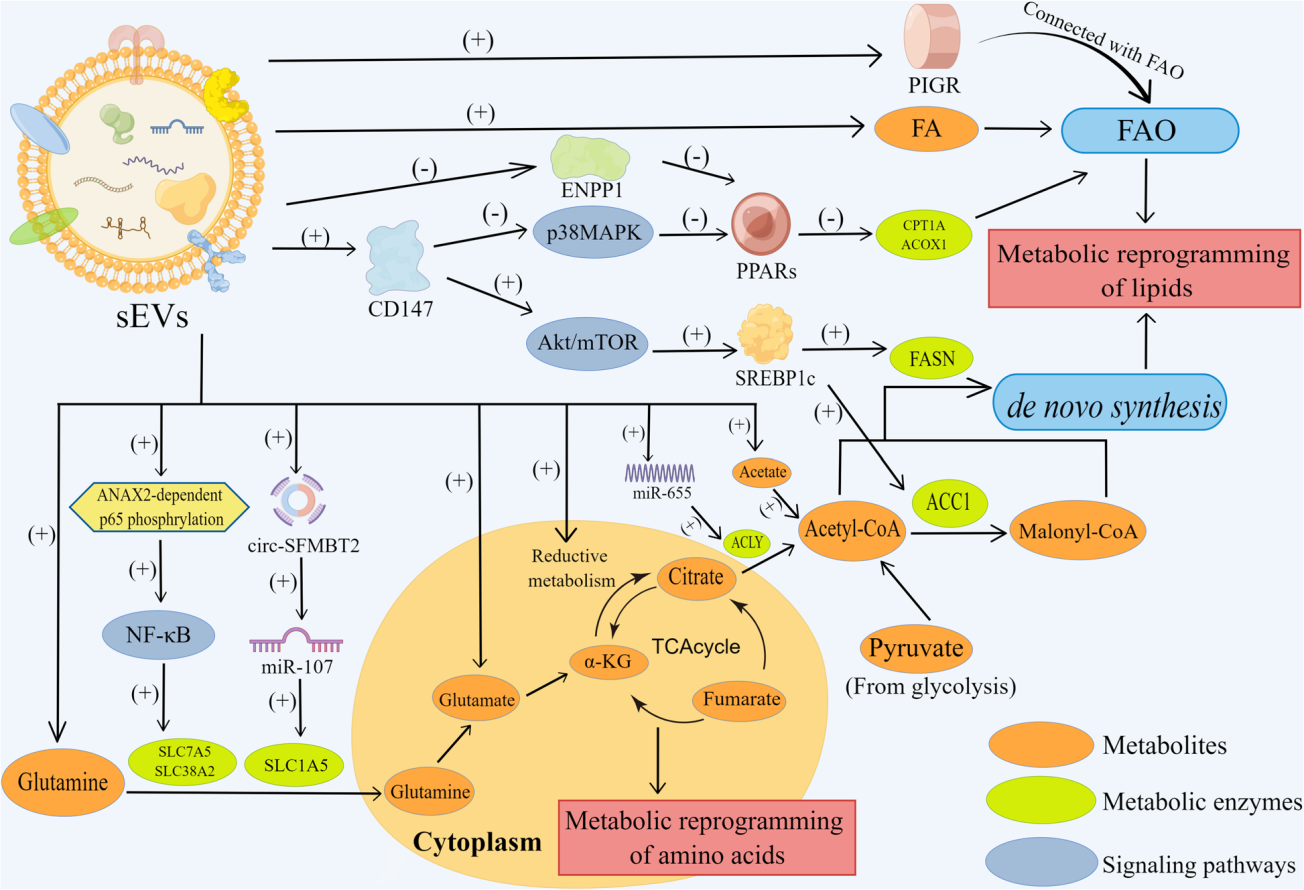
The potential mechanisms of sEV-mediated metabolic reprogramming of amino acids and lipid metabolism. The figure was created using Figdraw (www.figdraw.com). ACLY: ATP citrate lyase; FA: fatty acid; XIST: X inactive specific transcripts; ACC: Acetyl-CoA carboxylase; FASN: Fatty acid synthase; SREBPs: Sterol regulatory element binding proteins; FAO: Fatty acid oxidation; PIGR: Polymerized immunoglobulin receptor; PPARs: Peroxisome proliferator-activated receptors; CPT1A: Carnitine palmitoyltransferase 1; ACOX1: Acyl-coenzyme aoxidase 1; circ-SFMBT2: circRNA Scm-like with four malignant brain tumor domains 2; ENPP1: Ectonu nucleotide pyrophosphatase/phosphodiesterase 1

Except for aerobic glycolysis, lipid synthesis has been recognized as another prominent metabolic abnormality essential for carcinogenesis [30]. Cancer cells obtain lipid molecule by virtue of two major mechanisms: the internalization of exogenic lipids from local environment and de novo lipogenesis of endogenic lipids [31]. Contrary to normal cells utilizing exogenous fatty acids (FA), proliferating tumor cells tend to supplement FA via de novo lipogenesis [32]. Like glycolysis, sEV-mediated cancer cell metabolic reprogramming is realized by affecting crucial enzymes associated with de novo lipogenesis (Fig. 2).

#### sEVs affect lipid anabolism in cancer

ATP citrate lyase (ACLY) is the first rate-limiting enzyme participating in the production of new FA, linking glucose and FA metabolism by transforming citrate to oxaloacetic acid and acetyl-CoA, which enters cytoplasmic FA synthesis [33]. sEV lncRNA X inactive specific transcripts (XIST) promotes cells proliferation and progression in osteosarcoma tissues through a novel miR-655/ACLY signaling. In regard to the mechanism, XIST was shown to down-regulate the miR-655 expression, bringing about the increased ACLY levels, thereby increasing the lipid deposition in cancer cells [34]. Acetyl-CoA carboxylase (ACC) and fatty acid synthase (FASN) are major lipogenic enzymes in lipid anabolism that were observed to be enhanced by CD147, a transmembrane glycoprotein enriched in sEVs from patients with hepatocellular carcinoma (HCC) [35, 36]. Lipogenic enzyme genes including ACC and FASN are trans-activated by sterol regulatory element binding proteins (SREBPs) in the regulation of FA synthesis [37]. Furthermore, sEV CD147 promotes the expression levels of SREBP1c by stimulating the Akt/mTOR signaling route, which, in turn, activates the transcription of lipogenic genes FASN and ACC1 to enhance de novo lipogenesis [36].

Acetyl-CoA is a precursor of FA synthesis that is derived primarily from glucose under the conditions of adequate nutrition and oxygen. Nevertheless, in hypoxia, glucose-derived pyruvate is shunted to produce lactic acid instead of being used to produce acetyl-CoA, thus affecting the synthesis of FA, but the contribution rate of glutamine to acetyl-coA is increased to make up for the production of acetyl-CoA [38]. sEVs from cancer associated fibroblasts (CAFs) shift the carbon source from the oxidative glucose pathway to reductive glutamine metabolism, which can generate α-ketoglutarate to produce citrate, increasing acetyl-CoA synthesis in tumor cells [38]. Acetate is a significant fountain of acetyl-CoA, especially faced with the absence of oxygen [39]. CAF-derived sEVs carried numerous acetates to facilitate the contribution of acetate to promote FA synthesis, allowing cancer cells acquire enough energy to grow and metastasize [38]. These results demonstrated that sEVs rewrite cancer cells de novo lipogenesis by influencing the related enzymes.

#### sEVs influence the catabolism of lipids in cancer

Unlike the definite FA synthesis pathways, FA oxidation in tumor cells (FAO; also known as beta oxidation) remains unknown to a great extent [32]. sEVs from adipocytes transferred FAO-related proteins (including ECHA, HCDH, HCD2, etc.) and FAO substrate (FA) to melanoma cells, driving FAO to realize metabolic remodeling in tumor [40]. Polymerized immunoglobulin receptor (PIGR) is an sEV-associated glycoprotein, whose increased expression is concerned tightly with liver metastasis and poor prognosis in patients with colon cancer [41]. Abnormally expressed PIGR in CRC may be comprised in the modulation of signal pathways related to the FA metabolism, such as mitochondrial FA beta oxidation, thus reprogramming cancer cells metabolically [42]. Peroxisome proliferator-activated receptors (PPARs) are members of the nuclear receptor superfamily that are responsible for fat and carbohydrate metabolism and homeostasis and are expressed in many human solid tumors [43]. Carnitine palmitoyltransferase 1 (CPT1A) and acyl-coenzyme aoxidase 1(ACOX1) are the targets of PPARs and critical enzymes involved in FAO pathway. sEV CD147 could suppress FAO by suppressing p38 MAPK signaling to down-regulate the expressions of PPARα and its target genes of CPT1A and ACOX1, which consequently reprogrammed cancer cell lipid metabolism except for its functions in de novo lipogenesis [36]. Ectonu nucleotide pyrophosphatase/phosphodiesterase 1(ENPP1) is a type II transmembrane glycoprotein, a decrease in which is connected with poor prognosis in HCC patients [44, 45]. Down-regulated sEV-associated ENPP1 and FA fatty acid degradation and PPAR signaling pathways are the signaling pathways for the enrichment of sEV ENPP1 co-expression molecules [46]. Additionally, ENPP1 may take part in the supervision of other lipid metabolic routes, including FA ω-oxidation [45]. The above studies confirmed that sEVs reprogram cancer metabolism in the anabolism and catabolism of lipids through their cargos, thus promoting the occurrence and development of tumors.

### sEVs regulate amino acid metabolic reprogramming in cancer cells

Improved metabolism of glutamine is a common metabolic change in tumors, and its importance only second to glucose in cancer metabolism [33]. sEVs play a critical role in amino acid metabolism changes by transferring various molecules. Glutamine is transported to the cytoplasm by glutamine transporters, and the catabolism of glutamine begins with the conversion of glutamine to glutamate. sEVs from BMSCs contain glutamic acid and lactic acid, which can directly provide nutrition for cancer cells and promote the malignant development in breast cancer [47]. sEVs from CAFs may supply a “ready” metabolite cargo such as glutamine and arginine to promote cancer progression in nutrient-poor conditions [38]. The CAF-derived sEV lncC01614 mediates enhanced glutamine absorption in cancer cells by interacting with ANXA2 and p65 to promote ANXA2-dependent p65 phosphorylation, which, in turn, activates the NF-κB pathway to increase glutamine transporters SLC38A2 and SLC7A5, ultimately enhancing the inflow of glutamine into tumor cells [48]. SLC1A5 is another glutamine transporter that works as a cancer-promoting factor in esophageal cancer (EC). Exosome circRNA Scm-like with four malignant brain tumor domains 2 (circ-SFMBT2) use the miR-107/SLC1A5 cascade to induce the malignant phenotype of EC. Mechanistically, circ-SFMBT2 reduced the consumption of glutamine and the synthesis of glutamate or α-ketoglutarate by downregulating miR-107 to increase the levels of SLC1A5 [49]. Glutamine can be reduced to α-ketoglutaric acid in chondriosomes as an intermediary in the TCA cycle and provide carbon for lipogenic acetyl-CoA in two different pathways. Cells can oxidatively metabolize glutamine-derived αKG in the TCA cycle and generate pyruvate [50]. However, hypoxic cells rely almost exclusively on reductive carboxylation of αKG to generate citrate for de novo lipogenesis [51]. In reductive glutamine metabolism, α-ketoglutaric acid generates M5 citrate, which is further catalyzed to M3 fumarate and M3 malate [38, 52]. The addition of CAF-derived sEV in cultured cancer cells increased the M3 fumarate and M3 malate in them, suggesting that sEVs enhanced glutamine entry into the TCA cycle through reductive pathway to change cancer cell metabolism [38]. Besides glutamine, arginine is the major amino acids critical for tumor cell division and sEVs from human amniotic epithelial cells (hAEC), which significantly modulated amino acid metabolism by fueling the Warburg effect and running out arginine to exert anti-cancer functions [53]. However, most of the studies mainly focus on glycolysis, and the mechanism of how sEVs influence the amino acid metabolism in cancer cells should be explored as an emerging development direction in the future.

### Metabolic reprogramming in cancer cells promotes sEV secretion

The oxygen demand of tumor quickly increases owing to their rapid growth and hypoxia is the critical characteristic of solid tumors [54]. The recent research shows that hypoxia enhances sEV secretion by tumor cells and delivers sEV cargos like miR-301a-3p, which can induce the M2 polarization of macrophages and accelerate tumor progression [55, 56]. As a critical pathway in hypoxia, the function of HIF-1 can be elevated by metabolic changes in tumor cells, which stimulates the gene expressions of glycolysis-related proteins including glucose transporters and glycolytic enzymes, which absorb glucose and transform it into lactate in the rewriting of cancer metabolism [57]. The lactate from tumor glycolysis creates an acidic environment and facilitates the release of sEVs due to their acidophilic nature [58]. In brief, the metabolic changes of cancer cells may increase the secretion of tumor-derived sEVs carrying different molecules mentioned before, which speed up tumor metastasis.

## sEV-mediated metabolic reprogramming of the TME communicates cancer cells and stromal cells

sEVs carry biologically active substances to shuttle between tumor cells and stromal cells in the TME, consequently affecting tumor progression. The feedback loop between stromal cells and cancer cells facilitates the growth of cancer cells by altering metabolism via sEVs [3] (Table 1).

**Table 1:** Overview of sEV-mediated metabolic reprogramming in the TME

sEVs cargoes	Donor cells	Recipient cells	Function	Ref
ITGB4	Triple-negative breast cancer cells	CAFs	Induces autophagy and the production of lactic acid in CAFs	[59]
TGFβ	Bladder cancer cells		Promotes the transformation of fibroblasts to CAFs	[60]
miR-105	Breast cancer cells		Reprograms metabolic patterns of CAFs to fuel cancer cells	[61]
LMP1	CM cells		Activates autophagy and glycolysis in CAFs	[62]
HSPC111	Colorectal cancer cells		Reprograms lipid metabolism in CAFs	[63]
NME1 /2	Invasive breast carcinoma cells		Modify Lipid metabolism in Fibroblasts	[64]
miR-155, miR-210	Melanoma cells		Increases glycolysis and decreases OXPHOS in fibroblasts	[65]
SNHG3	CAFs	Breast cancer cells	Inhibits OXPHOS and increases glycolysis in tumor	[25]
Amino acids, lipids		Prostate and pancreatic cancer cells	Directly provide metabolites to cancer cells	[38]
PKM2	Hypoxic lung cancer cells	Macrophages	Induces M2 macrophage polarization	[66]
HMGB-1	Lung cancer cells		Enhances glycolysis to induce immunosuppressive macrophages	[67]
HISLA	TAMs	Breast cancer cells	Promotes the glycolysis and apoptotic resistance of cancer cells	[16]
miR-155	Breast cancer cells	CAAs	Promotes beige/brown differentiation and revise metabolic characteristics in adipocytes	[68]
AM	Pancreatic cancer cells		Promotes adipocyte lipolysis	[69]
FAO enzymes and FA	CAAs	Melanoma cells	Stimulate FAO in order to enhance aggressiveness of cells	[40]
miR-100	MSCs	Colorectal cancer cells	Inhibits cancer cells glycolysis to induce the apoptosis	[28]
Lactic acid and glutamic acid		Breast cancer cells	Support tumor growth	[47]
PKM2	Hepatocellular carcinoma cells	Monocytes	Leads to monocyte-to-macrophage differentiation and tumor microenvironment remodeling	[70]
VEGF/VEGFR	Acute myeloid leukemia cells	ECs	Enhances ECs glycolysis and proliferation	[71]
CAT1	Colorectal cancer cells		Supports amino acid metabolism in ECs	[72]

### sEV-mediated metabolic reprogramming connects CAFs with tumor cells

CAFs are well accepted as critical part of the abundant stromal cells located in the TME [73]. CAFs regulate cancer progression by stimulating cell proliferation, promoting immune escape, and stimulating angiogenesis and resistance to treatment [74]. CAFs can modify the metabolism of cancer cells by undergoing aerobic glycolysis to produce high fuels (like lactic acid) to feed cancer cells so that their proliferation ability can be enhanced. The metabolic cooperation phenomenon is the ‘reverse Warburg effect’, being similar to the ‘Warburg effect’ in tumors [75]. CAFs with the catabolic phenotype generate and release a quantity of lactate through monocarboxylate transporter 4(MCT4), while neighboring tumor cells could enhance lactate uptake through MCT1 and oxidize it to supply energy [76]. These results suggest the existence of tight metabolic junctions between fibroblasts and tumor cells.sEVs have been proven to create a shared metabolic environment and mediate important communication between CAFs and cancer cells. Triple-negative breast cancer cell-derived sEVs provide ITGB4 protein to CAFs, thus inducing autophagy and the production of lactic acid in CAFs [59]. Bladder cancer-derived sEVs mediate the process of TGFβ transportation to normal fibroblasts, thereby promoting the transformation of fibroblasts to CAFs [60]. Besides, the tumor-derived exosome miR-105 makes CAFs appear in different metabolic patterns when faced with high or low nutrient levels, by activating MYC signaling. Glucose and glutamine metabolisms are increased in CAFs to feed contiguous cancer cells when nutrient levels are high and these CAFs are capable of transforming metabolic wastes into energy-rich metabolites when lacking nutrition [61]. Apart from glucose metabolism, sEV-mediated metastasis suppressors NME1 and NME2 downregulate the expression of FA and cholesterol metabolism-related genes meaningfully to modify lipid metabolism in fibroblasts [64].

Meanwhile, CAF-derived sEVs are also dominant contributors to metabolic remodification in cancer cells. In breast cancer, CAF-derived sEV lncRNA SNHG3 was found to up-regulate the levels of PKM1/M2 as a molecular sponge of miR-330-5p, thus suppressing OXPHOS and enhancing the tumor cell proliferation [25]. Furthermore, CAF-derived sEVs shift the carbon source from the oxidative glucose pathway to glutamine with the help of the reductive carboxylation pathway in the TCA cycle, boosting the level of glutamine in tumor cells [38]. Intra-exosome metabolomics also shows that CAF-derived sEVs provide substances like amino acids, lipids, and TCA-cycle intermediates for tumor cells to realize metabolic reprogramming [38]. In conclusion, sEV-associated metabolic modification is a crucial means of intercellular communications between cancer cells and CAFs, promoting tumor cell growth.

### sEV-mediated metabolic reprogramming triggers macrophage polarization

During pro-tumor inflammation, macrophages have been reported to play a vital role [8]. Typically, macrophages are divided into two main phenotypes on the basis of their functions—the M1 phenotype manifesting immunostimulatory peculiarities and M2 phenotype manifesting immunosuppressive peculiarities [77]. Accumulating evidence suggests that as major constitutions of the TME, TAMs in tumor cells mainly exist as an M2-like phenotype [78]. Macrophage polarization depends on comprehensive intracellular metabolic alteration and usually, M1 macrophages are supported by aerobic glycolysis, while the M2 phenotype relies on OXPHOS [79]. Many experiments attempted to analyze the connection between macrophage polarization and sEVs in tumors. Hypoxic tumor-derived sEV PKM2 was reported to trigger M2 polarization of macrophages via the AMPK pathway to promote lung cancer progression and metastasis [66]. A study showed that although TAMs typically act as M2-like macrophages, their metabolic features are different from these cells. Instead, being analogous to M1 macrophages to a large extent, TAMs perform glycolytic dominant metabolism to survive and sustain their functions [79]. Tumor-derived sEVs increase macrophage glycolysis through TLR2-NF-kB signaling to make them obtain immunosuppressive phenotype, which was distinguished by abundant (programmed cell death ligand-1) PD-L1 expression and PD-L1 was positively relevant with levels of GLUT-1 from primary tumors [67]. TAMs also promote the glycolysis and apoptotic resistance in cancer cells through sEVs transferring HISLA to block the degradation of HIF-1α. In contrast, glycolytic cancer cells release lactate to upregulate HISLA in TAMs to form a feed-forward loop [16]. Both these studies demonstrated that sEVs play a key role in the metabolic reshaping of TAMs and cancer cells, promoting macrophage polarization and tumor progression.

### sEV-mediated metabolic reprogramming stimulates CAA oxidation

In light of the fact that obesity is linked to cancer progression, adipocytes are recognized as key players in the TME [80]. Cancer-associated adipocytes (CAAs) produce adipokines, and adipocytokines to influence the migration, and growth of cancer cells [81]. In contrast, tumor secretions trigger lipolysis in nearby adipocytes, which is called cachexia [82]. When referring to tumors, massive delipidation occurs in CAAs, which can provide FA for the fueling of FAO in tumor cells [80]. sEVs are involved in the bidirectional metabolic cross-talk between CAAs and cancer cells. Breast cancer-derived sEVs can secret miR-155 to promote beige/brown differentiation and revise metabolic characteristics in resident adipocytes through the down-regulation of PPARγ expression [68]. Adipocyte‐derived sEVs stimulate melanoma FAO to enhance the aggressiveness of cells with melanoma by providing FAO enzymes and FA [40]. Besides, PC cells secrete sEV adrenomedullin to motivate ERK1/2 and p38 MAPK axis, thereby promoting adipocyte lipolysis associated with early weight loss in PC patients [69]. Accordingly, it can be speculated that sEVs derived from adipocytes could enhance tumor development by reshaping cancer cell lipids metabolism.

### sEV-mediated metabolic reprogramming mediate MSCs and tumor cell communication

MSCs are multipotent cells, generally presented in tissues throughout the human body. In a TME, MSCs are educated as tumor-promoting phenotypes by tumoral factors, developing into cancer-associated mesenchymal stem cells (CA-MSCs) [83]. Specially, MSCs are capable of differentiating into CAAs as well as CAFs to affect the metabolism of cancer cells [83]. MSCs can evolve into CA-MSCs via microenvironment acidification arisen by the glycolysis of tumors [84]. In an HCC-mimicking microenvironment, MSCs glycolysis activity is enhanced because of mitochondrial dysfunction [47]. sEV-involved metabolic reprogramming features in the interaction between tumor cells and MSCs. MSC-derived sEVs secrete miR-100 to upregulate the expression of miR-143, thus repressing mTOR signaling, which can lead to the suppression of its downstream signaling molecule including glycolysis enzyme HK2 to inhibit glycolysis [28]. Excluding the impacting signaling pathway, MSC-derived sEVs directly support tumor progression by providing metabolic substrates including lactic acid and glutamic acid to cancer cells [47]. In conclusion, sEV-mediated metabolic reprogramming serves as a communication system between MSCs and tumor cells.

## sEV-mediated metabolic reprogramming in forming pre-metastatic niches and promoting metastasis

**Fig. 3:**
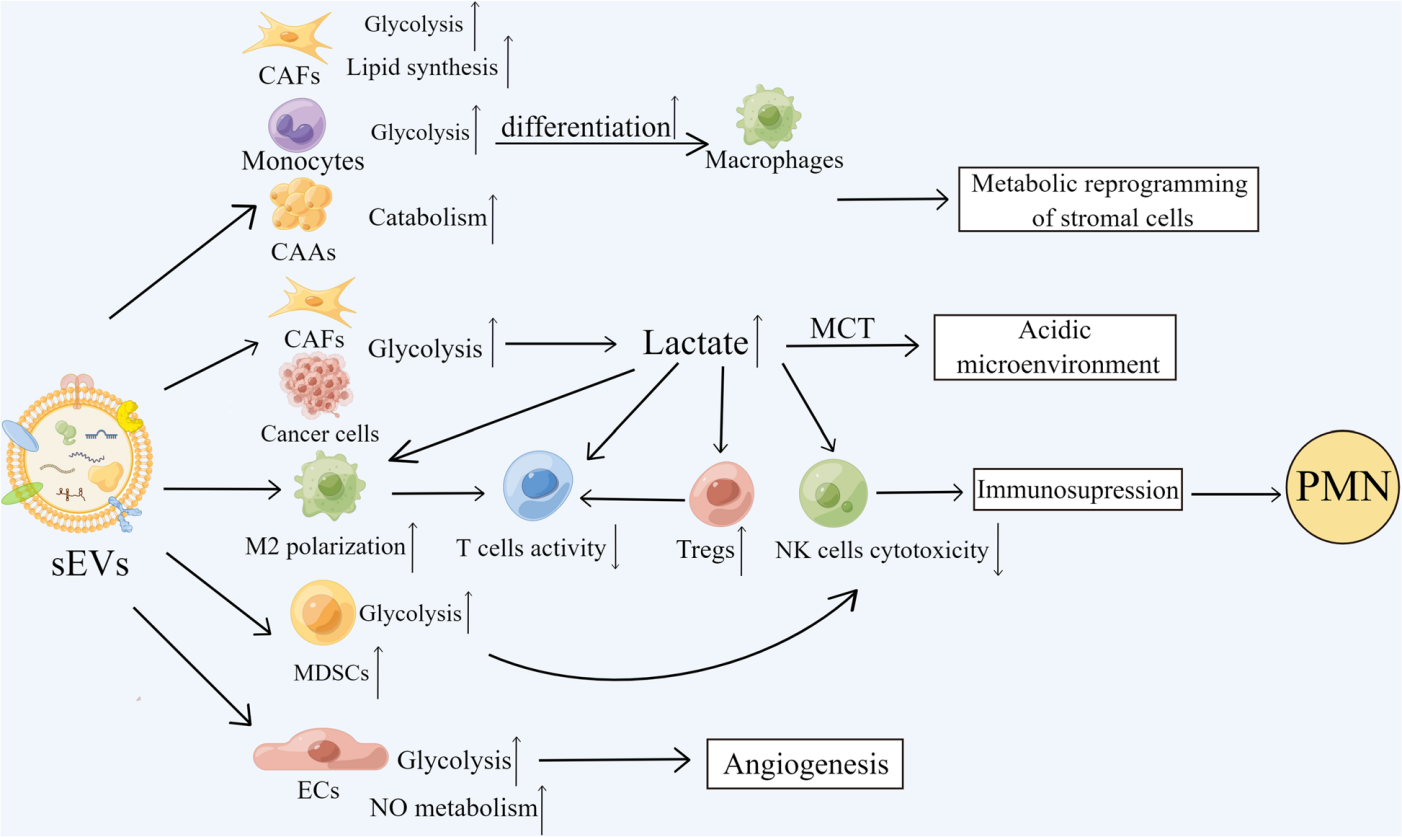
The functions of sEV-mediated metabolic reprogramming in the formation of a PMN. The figure was created using Figdraw (www.figdraw.com). PMN: The pre-metastatic niche; CAFs: Cancer-associated fibroblasts; CAAs: Cancer-associated adipocytes; Tregs: Regulatory cells; NK cells: Natural killer cells; MDSCs: Myeloid-derived suppressor cells; MCT: Monocarboxylate transporter; ECs: Endothelial cells

Metastasis is the hallmark of cancer, being the continuous challenge in cancer therapy resistance and mortality. Before cancer cells successfully disseminate to secondary organs, the tumor metastasis supporting environment, termed as the ‘pre-metastatic niche’ has already formed [8]. sEV-mediated metabolic reprogramming motivates the creation of advantageous microenvironment for tumor metastasis by being involved in critical procedures such as angiogenesis and immunosuppression (Fig. 3).

### Shaping acidic tumor microenvironment

In cancer glycolysis, glucose is metabolized to lactate and it can be exported to the extracellular space through MCT, finally forming a high lactic acid TME [85, 86]. sEVs from hAEC could enhance the lactate secretion by potentiating the glycolysis pathway and inducing excessive lactate formation in cancer cells [53]. Except for cancer cells, the reverse Warburg effect of CAFs contributes to the formation of acidic microenvironment, and high-glycolytic-activity fibroblasts could release lactic acid via MCT1 to the TME [87]. Exposure of fibroblasts to melanoma-derived sEVs is the reason for the enhanced glycolysis and reduced OXPHOS in these cells, which consequently induces the acidic microenvironment [65]. The low pH produced via lactic acid by modifying glucose/glutamine metabolism is conducive to the existence of immunosuppressive cells in the PMN, leading to tumors escaping from immune surveillance and the increased survival ability of tumor cells under high pressure [47, 86]. Besides, lactate can induce macrophages to overexpress vascular endothelial growth factor (VEGF), hence producing more blood vessels beneficial for the epithelial–mesenchymal transition of tumors [86]. The acidification of TME has been demonstrated to exacerbate the metastasis of cancer cells [88]. Another experiment proposed that local acidification of stroma was conducive to the PMN formation [65]. Thus, the effects of sEVs promoting lactate production to shape acidic matrix pave the way for the PMN establishment.

### Angiogenesis

Angiogenesis, a complicated procedure whereby tumors obtain plentiful nutrients, is fundamental for tumor progression and finally promotes metastasis as a dominant hallmark of the PMN [89]. sEVs carrying multiple pro-angiogenic factors promote angiogenesis in physiological and pathological conditions [90]. Recent research has evidenced that the crosstalk between autophagy and sEV secretion could influence angiogenesis by mediating the function of human ECs [91, 92]. Cancer-associated sEV is one of the potential mechanisms that accelerate vessel formation [58]. sEVs could induce phenotypic and functional changes in ECs. Potentially, the affluent cargo including RNAs, miRNAs, and angiogenic proteins seems to be effective in phenotypic modulation of ECs and in contributing to angiogenesis [93]. Over the last few years, ECs have been reported to depend on aerobic glycolysis rather than oxidative phosphorylation to generate ATP during angiogenesis. The unique metabolic feature of ECs is crucial for tumor cell proliferation and migration [94]. VEGF is a well-known angiogenesis-related factor by which EC metabolism can be transformed from OXPHOS to the glycolysis pathway [95]. AML-derived sEVs contain VEGF, VEGF receptor (VEGFR) messenger RNA, thus modulating VEGFR expression in ECs to enhance its glycolysis and proliferation [71]. Except for glucose metabolism, sEVs are able to carry high-affinity cationic amino acid transporter 1, which is identified as the main shipper of arginine to support arginine transfer and NO metabolism, hence promoting EC growth [72]. EVs were found to drive the formation of PMN by stimulating angiogenesis [94].

### Metabolically reprogramming stromal cells

sEVs could reprogram stroma cell metabolism especially CAFs in the PMN to accelerate metastasis. In breast cancer, Tumor-derived sEVs transport miR-122 to decrease the glycolytic utilization in matrix cells by targeting PKM2, making tumor cells at pre-metastatic sites use glucose to the maximum extent [96]. Moreover, CRC cell-derived sEV HSPC111 promotes PMN establishment by phosphorylating ACLY, which can reprogram lipid metabolism in CAFs [63]. sEVs miR-155 and miR-210 released by melanoma cells led to an increase in aerobic glycolysis and decrease in OXPHOS in fibroblasts to favor PMN formation [65]. Except for CAFs, metabolic changes have taken place in other important stromal cells well. sEV PKM2 induced metabolic reshaping in monocytes and STAT3 phosphorylation in the nucleus to overexpress differentiation-associated transcription factors, resulting in monocyte-to-macrophage differentiation and TME remodeling, eventually promoting tumor progression [70]. Wu et al. [97] found that sEVs from the tumor-adipocyte coupling provoked beige/brown differentiation and remodeled adipocytes metabolically to enhance metastasis. Additionally, sEVs carrying VEGF were reported to enhance EC glycolysis [98]. Altogether, noncancerous cell metabolism has been reprogrammed by rapidly internalizing sEVs secreted by cancer cells, making metabolic reprogramming the critical contributor to form PMN and exacerbate metastasis.

### Immunosuppression

Immunosuppression may be the most significant characteristic of the whole features of the PMN [99]. As mentioned above, sEVs could stimulate lactate production and extracellular acidification arisen from lactate secretion, which could affect immune cells (natural killer cells [NK cells], effector T cells) and immunosuppressive cells (M2, regulatory cells [Tregs] and myeloid-derived suppressor cells [MDSCS]), building the immunosuppressive network in cancer cells [100]. Lactate could straight suppress the cytolytic activity of NK cells and mediately suppress their effects via raising the amounts of MDSCs, which hinder NK cytotoxicity [101]. PD-L1/programmed cell death receptor-1 (PD-1) signaling is classic tumor immune escape mechanism. M2-type macrophages are mostly engaged in immunosuppression because their overexpressed PD-L1 could bind with PD-1 on effector T cells to stop the function of T cells and abundant lactate in the TME caused by sEVs could induce M2-type polarization [86, 102]. Also, tumor-derived sEVs could polarize macrophages into an immunosuppressive phenotype within the PMN through TLR2-NF-kB-dependent, glycolytic dominant metabolic rewriting by increasing the expression of PD-L1 in TAMs and lactate produced via glycolysis could act on NF-κB, further driving PD-L1 expression [67]. In an acidic TME, owing to the suppression of MCT-1 on T cells, T cells cannot banish accumulated lactate, causing suppression of function and metabolism [103]. However, for Tregs, which can obstruct other immune cells like T cells, high concentration of lactic acid facilitates its proliferation, finally promoting tumor immune escape [86]. In brief, sEVs could create an immunosuppressive environment by increasing lactic acid to influence the functions of various immune-related cells. sEVs originated from pancreatic ductal adenocarcinoma cells with the missing SMAD4 contribute to the MDSCs proliferation via increased glycolysis and calcium flux by means of transporting differentially expressed miRNA and protein relevant to SMAD4 [104]. Consequences of the above all demonstrated that the potential effect of exosome-related metabolic reprogramming in immunosuppression of the PMN. In sum, secretions from sEVs function as tumor-supportive substances by promoting the establishment of the PMN via metabolic modification.

## The emerging application of sEV-mediated metabolic reprogramming

### As diagnostic and prognostic markers

sEVs are readily available in almost all body fluids, including blood, urine, saliva and ascites. They can serve as a “liquid biopsy” allowing for non-invasive tumor real-time monitoring due to enriched bioactive molecules that reflect the pathologic state of the originating cell, thus providing a rich source of biomarkers [105, 106] (Table 2).

**Table 2:** Metabolism-associated sEVs from biofluids as cancer diagnostic biomarkers

Biomarkers	Cancer types	Biofluids	Clinical value	Ref
PKM1/2, ENO1, ALD, FBA	Ovarian cancer	plasma	Prediction of epithelial ovarian cancer recurrence	[107]
ciRS‐122	Colorectal cancer	serum	CiRS-122 overexpression is associated with chemoresistance	[23]
PKM2	Prostate cancer	serum	A novel clinical biomarker in PCa	[108]
circPDK1	Pancreatic cancer	serum	Diagnostic biomarker for early PC patients	[17]

The obvious increase in the four glycolysis-related proteins PKM1/2, enolase 1, aldolase and fructose-bisphosphate A in sEVs from patients with ovarian cancer recurrence circulating blood plasma can be used to predict cancer palindromia [107]. sEV circRNAs may be regarded as diagnostic and prognostic biomarker of tumors owing to their massive existence and significant effects in cancer progression [109]. Compared with the oxaliplatin-sensitive group, the expression level of ciRS-122, which could target PKM2 to promote glycolysis, was higher in the oxaliplatin-resistant patient serum sEVs, revealing that the level of ciRS‐122 was positively associated with CRC oxaliplatin resistance [23]. We can therefore accept ciRS‐122 mediating metabolism as a possible predictor of cancer drug resistance. Serum-derived sEV PKM2, a glycolytic enzyme, from patients with either primary prostate cancer (PCa) or metastasis managed to create a PMN, suggesting that exosome PKM2 can be regarded as a novel clinical biomarker in PCa [108]. High expression of circPDK1 in serum was linked with a poor prognosis of pancreatic cancer patients and it could stimulate c-Myc to enhance glycolysis. Thus, circPDK1 could be a novel diagnostic marker for early PC diagnosis [17]. These results demonstrate that the sEVs targeting metabolic reprogramming can be regarded as prospective diagnostic and prognostic biomarkers.

### As therapeutic targets

Therapy aimed at sEVs shall be now put on the agenda of cancer treatment in view of the emerging role of sEV-mediated metabolic reprogramming in tumor progression as described. CAF-derived sEVs carry SNHG3 to facilitate tumor proliferation through enhancing glycolysis. These metabolic tendencies in CAFs were reversed by transducing to SNHG3 knockdown cells with sh-SNHG3-expressing lentivirus [25]. Moreover, the sEV inhibitor, GW4869 could inhibit the glycolysis and activation of cells by blocking the secretion of sEVs [110]. GW4869 may alter the metabolic alterations in breast tumors, subsequently generating the inhibition of the cancer progression [25]. We can anticipate that sEVs exploit the potential advantages to the full in future tumor treatment someday.

### As drug-delivery systems

sEVs have the ability to circulate in the body via blood and avoid immune responses, and the unique characteristic of sEVs perforating the blood–brain barrier makes them function as drug-delivery systems targeting cancer cells [111, 112]. Hence, the direct engineering (loading sEVs with curative agents) and indirect engineering (modifying parental cells to produce functional sEVs) of sEVs pave a new way for drug delivery [113]. MSCs and dendritic cells are two major cell sources for the engineering use of sEVs, and sEVs from MSCs are ordinarily utilized for delivery systems [114]. However, under the condition of tumor, the use of cancer-derived sEVs may bring more benefits owing to their targeted homing and immunactivation ability [114]. The application of short interfering RNAs represents a milestone personalized medicine development, and sEVs can transport si‐ciRS‐122 to reverse resistance to oxaliplatin by suppressing the glycolysis pathway [23]. In view of this, we expect the engineering of sEVs and the use of tumor-derived sEVs loading various substances to inhibit tumor growth by regulating metabolism widely.

### Conclusions and perspectives

sEVs, the powerful mediators in cell-to-cell communication, alter the metabolism of cancer and stromal cells to enhance the growth of tumor. Furthermore, sEVs promote PMN formation in various aspects by mediating metabolic reprogramming. Hence, the engineering of sEVs and the therapy targeting sEV-mediated metabolic reprogramming are regarded as novel approaches in cancer clinical treatment. sEVs can be regarded as potential diagnostic or therapeutic targets in cancers and function as drug-delivery systems to target tumor cells. Future studies are recommended to solve the following questions.1) How to use reprogramming of amino acids and lipids mediated by sEVs to provide more fresh ideas for clinical treatment of cancers? 2) How to improve sEV separation and purification techniques to increase their yield and purity in large-scale clinical application? 3) How to increase the targeting effect of engineered sEVs to reverse the metabolic changes in recipient cells and prevent the formation of PMN?4) Does the engineering of plant-derived sEVs present more advantages than human EVs due to the advantage of easily acquiring from natural plants or not? The current study about sEV-mediated metabolic reprogramming in PMN ushers in a promising new chapter in the future therapies for cancers.

## Data Availability

Not applicable.

## Abbreviations

TME: Tumor microenvironment
PMN: Pre-metastatic niche
sEVs: Small extracellular vesicles
miRNA: MicroRNA
ECs: Endothelial cells
MSCs: Mesenchymal stem cells
PI3K/Akt: Phospho3-kinase/AKT
HIF-1: Hypoxia-inducible factor-1
HK2: Hexokinase 2
LDHA: Lactate dehydrogenase A
GLUT: Glucose transporter
PKM: Pyruvate kinase M
lncRNA: Long non-coding RNA
TAMs: Tumor-associated macrophages
HISLA: HIF-1α-stabilizing lncRNA
PC: Pancreatic cancer
STAT3: Signal transducer and activator of transcription 3
CRC: Colorectal cancer
SNHG3: Small nucleolar RNA host gene 3
OXPHOS: Oxidative phosphorylation
ALDOA: Aldolase A
mTOR: Mammalian target of rapamycin
BM-MSCs: Bone marrow mesenchymal stem cells
FA: Fatty acid
ACLY: ATP citrate lyase
XIST: X inactive specific transcripts
ACC: Acetyl-CoA carboxylase
FASN: Fatty acid synthase
SREBPs: Sterol regulatory element binding proteins
HCC: Hepatocellular carcinoma
CAFs: Cancer-associated fibroblasts
FAO: Fatty acid oxidation
PIGR: Polymerized immunoglobulin receptor
PPARs: Peroxisome proliferator-activated receptors
CPT1A: Carnitine palmitoyltransferase 1
ACOX1: Acyl-coenzyme aoxidase 1
ENPP1: Ectonu nucleotide pyrophosphatase/phosphodiesterase 1
EC: Esophageal cancer
circ-SFMBT2: circRNA Scm-like with four malignant brain tumor domains 2
MCT: Monocarboxylate transporter
PD-L1: Programmed cell death ligand-1
CAAs: Cancer-associated adipocytes
CA-MSCs: cancer-associated mesenchymal stem cells
VEGF: Vascular endothelial growth factor
VEGFR: VEGF receptor
NK cells: Natural killer cells
Tregs: Regulatory cells
MDSCs: Myeloid-derived suppressor cells
PD-1: Programmed cell death receptor-1
PCa: Prostate cancer

## References

[1] Steeg PS (2016). Targeting metastasis. Nat Rev Cancer.

[2] Liu Y, Cao X (2016). Characteristics and significance of the pre-metastatic niche. Cancer Cell.

[3] Tan S, Xia L, Yi P, Han Y, Tang L, Pan Q, Tian Y, Rao S, Oyang L, Liang J (2020). Exosomal miRNAs in tumor microenvironment. J Exp Clin Cancer Res.

[4] Belli C, Trapani D, Viale G, D'Amico P, Duso BA, Della Vigna P, Orsi F, Curigliano G (2018). Targeting the microenvironment in solid tumors. Cancer Treat Rev.

[5] Yuan Y, Li H, Pu W, Chen L, Guo D, Jiang H, He B, Qin S, Wang K, Li N (2022). Cancer metabolism and tumor microenvironment: fostering each other?. Sci China Life Sci.

[6] Hanahan D (2022). Hallmarks of cancer: new dimensions. Cancer Discov.

[7] Nikfarjam S, Rezaie J, Zolbanin NM, Jafari R (2020). Mesenchymal stem cell derived-exosomes: a modern approach in translational medicine. J Transl Med.

[8] Yang E, Wang X, Gong Z, Yu M, Wu H, Zhang D (2020). Exosome-mediated metabolic reprogramming: the emerging role in tumor microenvironment remodeling and its influence on cancer progression. Signal Transduct Target Ther.

[9] Rezaie J, Ahmadi M, Ravanbakhsh R, Mojarad B, Mahbubfam S, Shaban SA, Shadi K, Berenjabad NJ, Etemadi T (2022). Tumor-derived extracellular vesicles: The metastatic organotropism drivers. Life Sci.

[10] Nemati M, Singh B, Mir RA, Nemati M, Babaei A, Ahmadi M, Rasmi Y, Golezani AG, Rezaie J (2022). Plant-derived extracellular vesicles: a novel nanomedicine approach with advantages and challenges. Cell Commun Signal.

[11] Chen JQ, Russo J (2012). Dysregulation of glucose transport, glycolysis, TCA cycle and glutaminolysis by oncogenes and tumor suppressors in cancer cells. Biochim Biophys Acta.

[12] Jiang C, Zhang N, Hu X, Wang H (2021). Tumor-associated exosomes promote lung cancer metastasis through multiple mechanisms. Mol Cancer.

[13] Kim JW, Zeller KI, Wang Y, Jegga AG, Aronow BJ, O'Donnell KA, Dang CV (2004). Evaluation of myc E-box phylogenetic footprints in glycolytic genes by chromatin immunoprecipitation assays. Mol Cell Biol.

[14] Stubbs M, Griffiths JR (2010). The altered metabolism of tumors: HIF-1 and its role in the Warburg effect. Adv Enzyme Regul.

[15] Wang H, Wang L, Pan H, Wang Y, Shi M, Yu H, Wang C, Pan X, Chen Z (2020). Exosomes derived from macrophages enhance aerobic glycolysis and chemoresistance in lung cancer by Stabilizing c-Myc via the inhibition of NEDD4L. Front Cell Dev Biol.

[16] Chen F, Chen J, Yang L, Liu J, Zhang X, Zhang Y, Tu Q, Yin D, Lin D, Wong PP (2019). Extracellular vesicle-packaged HIF-1α-stabilizing lncRNA from tumour-associated macrophages regulates aerobic glycolysis of breast cancer cells. Nat Cell Biol.

[17] Lin J, Wang X, Zhai S, Shi M, Peng C, Deng X, Fu D, Wang J, Shen B (2022). Hypoxia-induced exosomal circPDK1 promotes pancreatic cancer glycolysis via c-myc activation by modulating miR-628-3p/BPTF axis and degrading BIN1. J Hematol Oncol.

[18] Zhang Q, Jeppesen DK, Higginbotham JN, Demory Beckler M, Poulin EJ, Walsh AJ, Skala MC, McKinley ET, Manning HC, Hight MR (2018). Mutant KRAS Exosomes Alter the Metabolic State of Recipient Colonic Epithelial Cells. Cell Mol Gastroenterol Hepatol.

[19] Mathupala SP, Ko YH, Pedersen PL (2009). Hexokinase-2 bound to mitochondria: cancer's stygian link to the "Warburg Effect" and a pivotal target for effective therapy. Semin Cancer Biol.

[20] Jiang K, Yang J, Guo S, Zhao G, Wu H, Deng G (2019). Peripheral circulating exosome-mediated delivery of miR-155 as a novel mechanism for acute lung inflammation. Mol Ther.

[21] Jiang S, Zhang LF, Zhang HW, Hu S, Lu MH, Liang S, Li B, Li Y, Li D, Wang ED (2012). A novel miR-155/miR-143 cascade controls glycolysis by regulating hexokinase 2 in breast cancer cells. Embo j.

[22] Liu AM, Xu Z, Shek FH, Wong KF, Lee NP, Poon RT, Chen J, Luk JM (2014). miR-122 targets pyruvate kinase M2 and affects metabolism of hepatocellular carcinoma. PLoS One.

[23] Wang X, Zhang H, Yang H, Bai M, Ning T, Deng T, Liu R, Fan Q, Zhu K, Li J (2020). Exosome-delivered circRNA promotes glycolysis to induce chemoresistance through the miR-122-PKM2 axis in colorectal cancer. Mol Oncol.

[24] Jiang Z, Hu H, Hu W, Hou Z, Liu W, Yu Z, Liang Z, Chen S (2021). Circ-RNF121 regulates tumor progression and glucose metabolism by miR-1224-5p/FOXM1 axis in colorectal cancer. Cancer Cell Int.

[25] Li Y, Zhao Z, Liu W, Li X (2020). SNHG3 functions as miRNA sponge to promote breast cancer cells growth through the metabolic reprogramming. Appl Biochem Biotechnol.

[26] Wang C, Xu J, Yuan D, Bai Y, Pan Y, Zhang J, Shao C (2020). Exosomes carrying ALDOA and ALDH3A1 from irradiated lung cancer cells enhance migration and invasion of recipients by accelerating glycolysis. Mol Cell Biochem.

[27] Fang R, Xiao T, Fang Z, Sun Y, Li F, Gao Y, Feng Y, Li L, Wang Y, Liu X (2012). MicroRNA-143 (miR-143) regulates cancer glycolysis via targeting hexokinase 2 gene. J Biol Chem.

[28] Jahangiri B, Khalaj-Kondori M, Asadollahi E, Purrafee Dizaj L, Sadeghizadeh M (2022). MSC-Derived exosomes suppress colorectal cancer cell proliferation and metastasis via miR-100/mTOR/miR-143 pathway. Int J Pharm.

[29] Hu J, Zhao W, Huang Y, Wang Z, Jiang T, Wang L (2019). MiR-1180 from bone marrow MSCs promotes cell proliferation and glycolysis in ovarian cancer cells via SFRP1/Wnt pathway. Cancer Cell Int.

[30] Swierczynski J, Hebanowska A, Sledzinski T (2014). Role of abnormal lipid metabolism in development, progression, diagnosis and therapy of pancreatic cancer. World J Gastroenterol.

[31] Beloribi-Djefaflia S, Vasseur S, Guillaumond F (2016). Lipid metabolic reprogramming in cancer cells. Oncogenesis.

[32] Simeone P, Tacconi S, Longo S, Lanuti P, Bravaccini S, Pirini F, Ravaioli S, Dini L, Giudetti AM: Expanding roles of de novo lipogenesis in breast cancer. Int J Environ Res Public Health 2021, 18(7):3575. 10.3390/ijerph18073575 PMC8036647 33808259

[33] Li Z, Zhang H (2016). Reprogramming of glucose, fatty acid and amino acid metabolism for cancer progression. Cell Mol Life Sci.

[34] Zhu G, Xia Y, Zhao Z, Li A, Li H, Xiao T (2022). LncRNA XIST from the bone marrow mesenchymal stem cell derived exosome promotes osteosarcoma growth and metastasis through miR-655/ACLY signal. Cancer Cell Int.

[35] Sun N, Zhang C, Lee YT, Tran BV, Wang J, Kim H, Lee J, Zhang RY, Wang JJ, Hu J, et al. HCC EV ECG score: an extracellular vesicle-based protein assay for detection of early-stage hepatocellular carcinoma. Hepatology. 2023;77(3):774–8. 10.1002/hep.32692 PMC9887095 35908246

[36] Li J, Huang Q, Long X, Zhang J, Huang X, Aa J, Yang H, Chen Z, Xing J (2015). CD147 reprograms fatty acid metabolism in hepatocellular carcinoma cells through Akt/mTOR/SREBP1c and P38/PPARα pathways. J Hepatol.

[37] Xu X, So JS, Park JG, Lee AH (2013). Transcriptional control of hepatic lipid metabolism by SREBP and ChREBP. Semin Liver Dis.

[38] Zhao H, Yang L, Baddour J, Achreja A, Bernard V, Moss T, Marini JC, Tudawe T, Seviour EG, San Lucas FA (2016). Tumor microenvironment derived exosomes pleiotropically modulate cancer cell metabolism. Elife.

[39] Kamphorst JJ, Chung MK, Fan J, Rabinowitz JD (2014). Quantitative analysis of acetyl-CoA production in hypoxic cancer cells reveals substantial contribution from acetate. Cancer Metab.

[40] Clement E, Lazar I, Attané C, Carrié L, Dauvillier S, Ducoux-Petit M, Esteve D, Menneteau T, Moutahir M, Le Gonidec S (2020). Adipocyte extracellular vesicles carry enzymes and fatty acids that stimulate mitochondrial metabolism and remodeling in tumor cells. Embo J.

[41] Liu F, Ye P, Bi T, Teng L, Xiang C, Wang H, Li Y, Jin K, Mou X (2014). COLORECTAL Polymeric immunoglobulin receptor expression is correlated with hepatic metastasis and poor prognosis in colon carcinoma patients with hepatic metastasis. Hepatogastroenterology.

[42] Liu Y, Hu Y, Deng L (2022). The underlying roles of exosome-associated PIGR in fatty acid metabolism and immune signaling in colorectal cancer. J Oncol.

[43] Mirza AZ, Althagafi II, Shamshad H (2019). Role of PPAR receptor in different diseases and their ligands: physiological importance and clinical implications. Eur J Med Chem.

[44] Roberts F, Zhu D, Farquharson C, Macrae VE (2019). ENPP1 in the regulation of mineralization and beyond. Trends Biochem Sci.

[45] Li Z, He Q, Peng J, Yan Y, Fu C (2022). Identification of downregulated exosome-associated gene ENPP1 as a novel lipid metabolism and immune-associated biomarker for hepatocellular carcinoma. J Oncol.

[46] Nikonorova IA, Wang J, Cope AL, Tilton PE, Power KM, Walsh JD, Akella JS, Krauchunas AR, Shah P, Barr MM (2022). Isolation, profiling, and tracking of extracellular vesicle cargo in Caenorhabditis elegans. Curr Biol.

[47] Vallabhaneni KC, Penfornis P, Dhule S, Guillonneau F, Adams KV, Mo YY, Xu R, Liu Y, Watabe K, Vemuri MC (2015). Extracellular vesicles from bone marrow mesenchymal stem/stromal cells transport tumor regulatory microRNA, proteins, and metabolites. Oncotarget.

[48] Liu T, Han C, Fang P, Ma Z, Wang X, Chen H, Wang S, Meng F, Wang C, Zhang E (2022). Cancer-associated fibroblast-specific lncRNA LINC01614 enhances glutamine uptake in lung adenocarcinoma. J Hematol Oncol.

[49] Chang Z, Fu Y, Jia Y, Gao M, Song L, Zhang W, Zhao R, Qin Y (2021). Circ-SFMBT2 drives the malignant phenotypes of esophageal cancer by the miR-107-dependent regulation of SLC1A5. Cancer Cell Int.

[50] DeBerardinis RJ, Mancuso A, Daikhin E, Nissim I, Yudkoff M, Wehrli S, Thompson CB (2007). Beyond aerobic glycolysis: transformed cells can engage in glutamine metabolism that exceeds the requirement for protein and nucleotide synthesis. Proc Natl Acad Sci U S A.

[51] Metallo CM, Gameiro PA, Bell EL, Mattaini KR, Yang J, Hiller K, Jewell CM, Johnson ZR, Irvine DJ, Guarente L (2011). Reductive glutamine metabolism by IDH1 mediates lipogenesis under hypoxia. Nature.

[52] Hensley CT, Wasti AT, DeBerardinis RJ (2013). Glutamine and cancer: cell biology, physiology, and clinical opportunities. J Clin Invest.

[53] Bolouri MR, Ghods R, Zarnani K, Vafaei S, Falak R, Zarnani AH (2022). Human amniotic epithelial cells exert anti-cancer effects through secretion of immunomodulatory small extracellular vesicles (sEV). Cancer Cell Int.

[54] Hu C, Chen M, Jiang R, Guo Y, Wu M, Zhang X (2018). Exosome-related tumor microenvironment. J Cancer.

[55] Kumar A, Deep G (2020). Hypoxia in tumor microenvironment regulates exosome biogenesis: molecular mechanisms and translational opportunities. Cancer Lett.

[56] Wang X, Luo G, Zhang K, Cao J, Huang C, Jiang T, Liu B, Su L, Qiu Z (2018). Hypoxic tumor-derived exosomal miR-301a mediates M2 macrophage polarization via PTEN/PI3Kγ to promote pancreatic cancer metastasis. Cancer Res.

[57] Shao X, Hua S, Feng T, Ocansey DKW, Yin L. Hypoxia-regulated tumor-derived exosomes and tumor progression: a focus on immune evasion. Int J Mol Sci 2022, 23(19):11789. 10.3390/ijms231911789 PMC9570495 36233088

[58] Mashouri L, Yousefi H, Aref AR, Ahadi AM, Molaei F, Alahari SK (2019). Exosomes: composition, biogenesis, and mechanisms in cancer metastasis and drug resistance. Mol Cancer.

[59] Sung JS, Kang CW, Kang S, Jang Y, Chae YC, Kim BG, Cho NH (2020). ITGB4-mediated metabolic reprogramming of cancer-associated fibroblasts. Oncogene.

[60] Ringuette Goulet C, Bernard G, Tremblay S, Chabaud S, Bolduc S, Pouliot F (2018). Exosomes induce fibroblast differentiation into cancer-associated fibroblasts through TGFβ signaling. Mol Cancer Res.

[61] Yan W, Wu X, Zhou W, Fong MY, Cao M, Liu J, Liu X, Chen CH, Fadare O, Pizzo DP (2018). Cancer-cell-secreted exosomal miR-105 promotes tumour growth through the MYC-dependent metabolic reprogramming of stromal cells. Nat Cell Biol.

[62] Wu X, Zhou Z, Xu S, Liao C, Chen X, Li B, Peng J, Li D, Yang L (2020). Extracellular vesicle packaged LMP1-activated fibroblasts promote tumor progression via autophagy and stroma-tumor metabolism coupling. Cancer Lett.

[63] Zhang C, Wang XY, Zhang P, He TC, Han JH, Zhang R, Lin J, Fan J, Lu L, Zhu WW (2022). Cancer-derived exosomal HSPC111 promotes colorectal cancer liver metastasis by reprogramming lipid metabolism in cancer-associated fibroblasts. Cell Death Dis.

[64] Mátyási B, Petővári G, Dankó T, Butz H, Likó I, Lőw P, Petit I, Bittar R, Bonnefont-Rousselot D, Farkas Z, et al. Extracellular vesicle-mediated metastasis suppressors NME1 and NME2 modify lipid metabolism in fibroblasts. Cancers (Basel) 2022, 14(16):3913. 10.3390/cancers14163913 PMC9406105 36010906

[65] Shu S, Yang Y, Allen CL, Maguire O, Minderman H, Sen A, Ciesielski MJ, Collins KA, Bush PJ, Singh P (2018). Metabolic reprogramming of stromal fibroblasts by melanoma exosome microRNA favours a pre-metastatic microenvironment. Sci Rep.

[66] Zhou S, Lan Y, Li Y, Li Z, Pu J, Wei L (2022). Hypoxic tumor-derived exosomes induce M2 macrophage polarization via PKM2/AMPK to promote lung cancer progression. Cell Transplant.

[67] Morrissey SM, Zhang F, Ding C, Montoya-Durango DE, Hu X, Yang C, Wang Z, Yuan F, Fox M, Zhang HG (2021). Tumor-derived exosomes drive immunosuppressive macrophages in a pre-metastatic niche through glycolytic dominant metabolic reprogramming. Cell Metab.

[68] Wu Q, Sun S, Li Z, Yang Q, Li B, Zhu S, Wang L, Wu J, Yuan J, Yang C (2018). Tumour-originated exosomal miR-155 triggers cancer-associated cachexia to promote tumour progression. Mol Cancer.

[69] Sagar G, Sah RP, Javeed N, Dutta SK, Smyrk TC, Lau JS, Giorgadze N, Tchkonia T, Kirkland JL, Chari ST (2016). Pathogenesis of pancreatic cancer exosome-induced lipolysis in adipose tissue. Gut.

[70] Hou PP, Luo LJ, Chen HZ, Chen QT, Bian XL, Wu SF, Zhou JX, Zhao WX, Liu JM, Wang XM (2020). Ectosomal PKM2 promotes HCC by inducing macrophage differentiation and remodeling the tumor microenvironment. Mol Cell.

[71] Wang B, Wang X, Hou D, Huang Q, Zhan W, Chen C, Liu J, You R, Xie J, Chen P (2019). Exosomes derived from acute myeloid leukemia cells promote chemoresistance by enhancing glycolysis-mediated vascular remodeling. J Cell Physiol.

[72] Ikeda A, Nagayama S, Sumazaki M, Konishi M, Fujii R, Saichi N, Muraoka S, Saigusa D, Shimada H, Sakai Y (2021). Colorectal cancer-derived CAT1-positive extracellular vesicles alter nitric oxide metabolism in endothelial cells and promote angiogenesis. Mol Cancer Res.

[73] Kanzaki R, Pietras K (2020). Heterogeneity of cancer-associated fibroblasts: Opportunities for precision medicine. Cancer Sci.

[74] Li C, Teixeira AF, Zhu HJ, Ten Dijke P (2021). Cancer associated-fibroblast-derived exosomes in cancer progression. Mol Cancer.

[75] Liang L, Li W, Li X, Jin X, Liao Q, Li Y, Zhou Y. 'Reverse Warburg effect' of cancer‑associated fibroblasts (Review). Int J Oncol 2022, 60(6):1. 10.3892/ijo.2022.5357 35425996

[76] Martinez-Outschoorn UE, Lisanti MP, Sotgia F (2014). Catabolic cancer-associated fibroblasts transfer energy and biomass to anabolic cancer cells, fueling tumor growth. Semin Cancer Biol.

[77] Eichmüller SB, Osen W, Mandelboim O, Seliger B. Immune modulatory microRNAs involved in tumor attack and tumor immune escape. J Natl Cancer Inst. 2017;109(10):djx034. 10.1093/jnci/djx034 28383653

[78] Choo YW, Kang M, Kim HY, Han J, Kang S, Lee JR, Jeong GJ, Kwon SP, Song SY, Go S (2018). M1 macrophage-derived nanovesicles potentiate the anticancer efficacy of immune checkpoint inhibitors. ACS Nano.

[79] Md-B N. Duncan-Moretti J, H Cd-C, Saldanha-Gama R, Paula-Neto HA, G GD, R LS, Barja-Fidalgo C. Aerobic glycolysis is a metabolic requirement to maintain the M2-like polarization of tumor-associated macrophages. Biochim Biophys Acta Mol Cell Res. 2020;1867(2): 118604. 10.1016/j.bbamcr.2019.118604 31760090

[80] Lazar I, Clement E, Attane C, Muller C, Nieto L (2018). A new role for extracellular vesicles: how small vesicles can feed tumors' big appetite. J Lipid Res.

[81] Cao Y (2019). Adipocyte and lipid metabolism in cancer drug resistance. J Clin Invest.

[82] Dirat B, Bochet L, Dabek M, Daviaud D, Dauvillier S, Majed B, Wang YY, Meulle A, Salles B, Le Gonidec S (2011). Cancer-associated adipocytes exhibit an activated phenotype and contribute to breast cancer invasion. Cancer Res.

[83] Atiya H, Frisbie L, Pressimone C, Coffman L (2020). Mesenchymal stem cells in the tumor microenvironment. Adv Exp Med Biol.

[84] Sarhadi VK, Daddali R, Seppänen-Kaijansinkko R: Mesenchymal stem cells and extracellular vesicles in osteosarcoma pathogenesis and therapy. Int J Mol Sci 2021, 22(20):11035. 10.3390/ijms222011035 PMC8537935 34681692

[85] Hirschhaeuser F, Sattler UG, Mueller-Klieser W (2011). Lactate: a metabolic key player in cancer. Cancer Res.

[86] Zhang Z, Hu Y, Chen Y, Chen Z, Zhu Y, Chen M, Xia J, Sun Y, Xu W (2022). Immunometabolism in the tumor microenvironment and its related research progress. Front Oncol.

[87] Galluzzi L, Kepp O, Kroemer G (2012). Reverse Warburg: straight to cancer. Cell Cycle.

[88] Rofstad EK, Mathiesen B, Kindem K, Galappathi K (2006). Acidic extracellular pH promotes experimental metastasis of human melanoma cells in athymic nude mice. Cancer Res.

[89] Hicklin DJ, Ellis LM (2005). Role of the vascular endothelial growth factor pathway in tumor growth and angiogenesis. J Clin Oncol.

[90] Shaban SA, Rezaie J, Nejati V (2022). Exosomes derived from senescent endothelial cells contain distinct Pro-angiogenic miRNAs and proteins. Cardiovasc Toxicol.

[91] Mahbubfam S, Rezaie J, Nejati V (2022). Crosstalk between exosomes signaling pathway and autophagy flux in senescent human endothelial cells. Tissue Cell.

[92] Hassanpour M, Rezaie J, Darabi M, Hiradfar A, Rahbarghazi R, Nouri M (2020). Autophagy modulation altered differentiation capacity of CD146+ cells toward endothelial cells, pericytes, and cardiomyocytes. Stem Cell Res Ther.

[93] Ludwig N, Yerneni SS, Razzo BM, Whiteside TL (2018). Exosomes from HNSCC promote angiogenesis through reprogramming of endothelial cells. Mol Cancer Res.

[94] Du W, Ren L, Hamblin MH, Fan Y: Endothelial cell glucose metabolism and angiogenesis. Biomedicines 2021, 9(2):147. 10.3390/biomedicines9020147 PMC7913320 33546224

[95] Potente M, Carmeliet P (2017). The link between angiogenesis and endothelial metabolism. Annu Rev Physiol.

[96] Fong MY, Zhou W, Liu L, Alontaga AY, Chandra M, Ashby J, Chow A, O'Connor ST, Li S, Chin AR (2015). Breast-cancer-secreted miR-122 reprograms glucose metabolism in premetastatic niche to promote metastasis. Nat Cell Biol.

[97] Wu Q, Li J, Li Z, Sun S, Zhu S, Wang L, Wu J, Yuan J, Zhang Y, Sun S (2019). Exosomes from the tumour-adipocyte interplay stimulate beige/brown differentiation and reprogram metabolism in stromal adipocytes to promote tumour progression. J Exp Clin Cancer Res.

[98] Rohlenova K, Veys K, Miranda-Santos I, De Bock K, Carmeliet P (2018). Endothelial cell metabolism in health and disease. Trends Cell Biol.

[99] Yang X, Zhang Y, Zhang Y, Zhang S, Qiu L, Zhuang Z, Wei M, Deng X, Wang Z, Han J (2021). The key role of exosomes on the pre-metastatic niche formation in tumors. Front Mol Biosci.

[100] Chen L, Huang L, Gu Y, Cang W, Sun P, Xiang Y: Lactate-lactylation hands between metabolic reprogramming and immunosuppression. Int J Mol Sci 2022, 23(19):11943. 10.3390/ijms231911943 PMC9569569 36233246

[101] Husain Z, Huang Y, Seth P, Sukhatme VP (2013). Tumor-derived lactate modifies antitumor immune response: effect on myeloid-derived suppressor cells and NK cells. J Immunol.

[102] Pu Y, Ji Q (2022). Tumor-associated macrophages regulate PD-1/PD-L1 immunosuppression. Front Immunol.

[103] Fischer K, Hoffmann P, Voelkl S, Meidenbauer N, Ammer J, Edinger M, Gottfried E, Schwarz S, Rothe G, Hoves S (2007). Inhibitory effect of tumor cell-derived lactic acid on human T cells. Blood.

[104] Basso D, Gnatta E, Padoan A, Fogar P, Furlanello S, Aita A, Bozzato D, Zambon CF, Arrigoni G, Frasson C (2017). PDAC-derived exosomes enrich the microenvironment in MDSCs in a SMAD4-dependent manner through a new calcium related axis. Oncotarget.

[105] Whiteside TL (2016). Tumor-derived exosomes and their role in cancer progression. Adv Clin Chem.

[106] Almohammai A, Rahbarghazi R, Keyhanmanesh R, Rezaie J, Ahmadi M (2021). Asthmatic condition induced the activity of exosome secretory pathway in rat pulmonary tissues. J Inflamm (Lond).

[107] Alharbi M, Lai A, Sharma S, Kalita-de Croft P, Godbole N, Campos A, Guanzon D, Salas-Burgos A, Carrion F, Zuñiga FA, et al. Extracellular vesicle transmission of chemoresistance to ovarian cancer cells is associated with hypoxia-induced expression of glycolytic pathway proteins, and prediction of epithelial ovarian cancer disease recurrence. Cancers (Basel) 2021, 13(14):3388. 10.3390/cancers13143388 PMC8305505 34298602

[108] Dai J, Escara-Wilke J, Keller JM, Jung Y, Taichman RS, Pienta KJ, Keller ET (2019). Primary prostate cancer educates bone stroma through exosomal pyruvate kinase M2 to promote bone metastasis. J Exp Med.

[109] Vahabi A, Rezaie J, Hassanpour M, Panahi Y, Nemati M, Rasmi Y, Nemati M (2022). Tumor Cells-derived exosomal CircRNAs: Novel cancer drivers, molecular mechanisms, and clinical opportunities. Biochem Pharmacol.

[110] Wan L, Xia T, Du Y, Liu J, Xie Y, Zhang Y, Guan F, Wu J, Wang X, Shi C (2019). Exosomes from activated hepatic stellate cells contain GLUT1 and PKM2: a role for exosomes in metabolic switch of liver nonparenchymal cells. Faseb j.

[111] Joo HS, Suh JH, Lee HJ, Bang ES, Lee JM. Current Knowledge and Future Perspectives on Mesenchymal Stem Cell-Derived Exosomes as a New Therapeutic Agent. Int J Mol Sci 2020, 21(3):727. 10.3390/ijms21030727 PMC7036914 31979113

[112] Rezaie J, Etemadi T, Feghhi M (2022). The distinct roles of exosomes in innate immune responses and therapeutic applications in cancer. Eur J Pharmacol.

[113] Rahbarghazi R, Jabbari N, Sani NA, Asghari R, Salimi L, Kalashani SA, Feghhi M, Etemadi T, Akbariazar E, Mahmoudi M (2019). Tumor-derived extracellular vesicles: reliable tools for cancer diagnosis and clinical applications. Cell Commun Signal.

[114] Rezaie J, Feghhi M, Etemadi T (2022). A review on exosomes application in clinical trials: perspective, questions, and challenges. Cell Commun Signal.

